# Divergence of Beauvericin Synthase Gene among *Fusarium* and *Trichoderma* Species

**DOI:** 10.3390/jof6040288

**Published:** 2020-11-15

**Authors:** Monika Urbaniak, Agnieszka Waśkiewicz, Grzegorz Koczyk, Lidia Błaszczyk, Łukasz Stępień

**Affiliations:** 1 Plant-Pathogen Interaction Team, Department of Pathogen Genetics and Plant Resistance, Institute of Plant Genetics of the Polish Academy of Sciences, Strzeszyńska 34, 60-479 Poznań, Poland; lste@igr.poznan.pl; 2 Department of Chemistry, Poznan University of Life Sciences, Wojska Polskiego 75, 60-625 Poznań, Poland; agat@up.poznan.pl; 3 Functional Evolution of Biological Systems Team, Department of Biometrics and Bioinformatics, Institute of Plant Genetics of the Polish Academy of Sciences, Strzeszyńska 34, 60-479 Poznań, Poland; gkoc@igr.poznan.pl; 4 Plant Microbiome Structure and Function Team, Department of Pathogen Genetics and Plant Resistance, Institute of Plant Genetics of the Polish Academy of Sciences, Strzeszyńska 34, 60-479 Poznań, Poland; lgol@igr.poznan.pl

**Keywords:** *Trichoderma*, *Fusarium*, cyclodepsipeptides, beauvericin

## Abstract

Beauvericin (BEA) is a cyclodepsipeptide mycotoxin, showing insecticidal, antibiotic and antimicrobial activities, as well as inducing apoptosis of cancer cell lines. BEA can be produced by multiple fungal species, including saprotrophs, plant, insect and human pathogens, particularly belonging to *Fusarium*, *Beauveria* and *Isaria* genera. The ability of *Trichoderma* species to produce BEA was until now uncertain. Biosynthesis of BEA is governed by a non-ribosomal peptide synthase (NRPS), known as beauvericin synthase (BEAS), which appears to present considerable divergence among different fungal species. In the present study we compared the production of beauvericin among *Fusarium* and *Trichoderma* strains using UPLC methods. *BEAS* fragments were sequenced and analyzed to examine the level of the gene’s divergence between these two genera and confirm the presence of active *BEAS* copy in *Trichoderma*. Seventeen strains of twelve species were studied and phylogenetic analysis showed distinctive grouping of *Fusarium* and *Trichoderma* strains. The highest producers of beauvericin were *F. proliferatum* and *F. nygamai*. *Trichoderma* strains of three species (*T. atroviride*, *T. viride*, *T. koningiopsis*) were minor BEA producers. The study showed beauvericin production by *Fusarium* and *Trichoderma* species and high variance of the non-ribosomal peptide synthase gene among fungal species from the *Hypocreales* order.

## 1. Introduction

**Figure 1:**
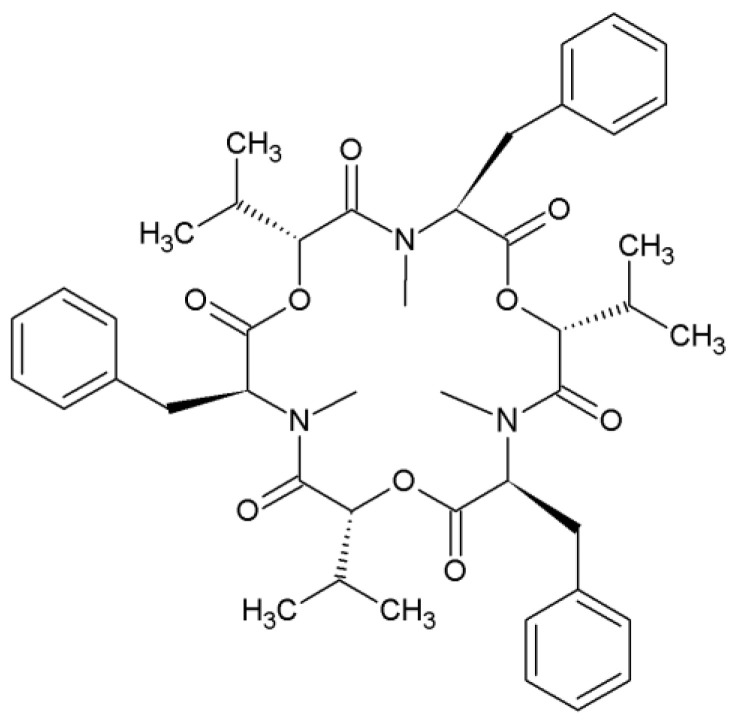
Chemical structure of beauvericin.

Beauvericin (BEA) is one of the main secondary metabolites from the cyclodepsipeptide group, consisting of three alternating D-2-hydroxyisovaleric (D-Hiv) acids and three *N*-methyl-L-phenylalanine residues (Figure 1) [1,2,3]. It has been proven in several research papers that BEA has many analogues, including naturally occurring beauvericins as well as precursor-directed beauvericins, which means that the corresponding amino acid precursor was added to the growing medium for their production [4,5,6,7,8].

BEA shows a wide range of biological activities, including insecticidal, antibiotic and antimicrobial activity against *Mycobacterium tuberculosis* and *Plasmodium falciparum* [5,9,10,11]. It can also be used as a co-drug with other antifungal compounds to treat fungal infections [8,12]. By activating calcium-sensitive cell apoptotic pathways, beauvericin induces apoptosis of cancer cell lines, hence, it can be used as a strong cytotoxic compound [7,9,10]. Because of its structural and ionophoric properties, this mycotoxin transports monovalent cations across the membranes and can be a free carrier that uncouples oxidative phosphorylation [2,13].

BEA biosynthesis by *Beauveria bassiana* was first reported by Hamill et al. [14]. The metabolite is also produced by other entomopathogenic fungi, such as *Isaria fumosorosea*, *I. farinosa*, *I. tenuipes*, to mention just a few [4,15,16], and also by phytopathogenic fungi such as *Fusarium*, belonging to the *Hypocreales* order [17,18,19,20,21,22]. On the other hand, *Trichoderma* species belong to this order, and *Trichoderma* fungi were not described earlier as beauvericin producers. However, the presence of the *BEAS* gene in the *Trichoderma* genome was reported [23].

Biosynthesis of BEA is assembled by the non-ribosomal peptide synthase (NRPS), known as beauvericin synthase (BEAS). BEAS with a molecular mass of approximately 250 kDa was described for the first time by Peeters et al. [24] in *Beauveria bassiana*. However, the beauvericin gene cluster ( bp, from *B. bassiana*), including a 9570 bp gene (*bbBeas*), encoding a putative cyclooligomer depsipeptide synthase (CODS), was reported by Xu et al. [11]. The molecular weight of bbBEAS (351,889 Da) designated by Xu et al. is higher by about 100 kDa than beauvericin synthase estimated earlier by Peeters et al. [24]. As for the *Fusarium* genus, a 9413 bp beauvericin synthase gene (*fpBeas*) was cloned and characterized for the first time by Zhang [25] and coworkers from *Fusarium proliferatum*.

As a whole, fungal NRPSs are large multidomain proteins (M = 347 kDa), organized in successive functional modules [11,25]. Each subsequent module is responsible for incorporating the proteinogenic and non-protein amino acids, along with carboxyl and hydroxyl acids, into the growing chain of the depsipeptide, which is eventually finalized as a mature cyclodepsipeptide [25]. While beauvericin synthase preferably accepts *N*-methyl-L-phenylalanine, the compound can be easily replaced by other hydrophobic amino acids such as leucine, norleucine, isoleucine, allylglycine and 2-amino-4-methylhex-4-enoic acid. Moreover, *ortho*-, *meta*- and *para*-fluoro derivatives of *N*-methyl-L-phenylalanine may be substituted in vitro [24].

A minimal module contains the three distinctly folded catalytic domains: (A) the adenylation domain responsible for recognition and activation of the substrate through adenylation with ATP, (T or PCP) thiolation or peptidyl carrier protein, which are involved in binding of the activated substrate to a 4′-phosphopantetheine (PP) cofactor through a thioester bond and transfer the substrate to the active sites of the last (C) condensation domain, were catalyzing the peptide bond (C-N) between the elongated chain and the activated amino acid is performed. Moreover, several other domains involved in chain construction have been identified, such as (M) methyltransferase, (E) epimerase, (Cy) heterocyclization and oxidation (Ox) domains, which modify the enzyme-bound precursors or extended peptide intermediates at various stages of the process. The (TE) thioesterase domain is responsible for the full-length NRPS product release by giving rise to free acids, lactones, or lactams [11,25,26,27].

In the present study, we compared the production of beauvericin among selected *Trichoderma* and *Fusarium* strains, concerning the comparative analysis of their partial *BEAS* homolog sequences, to gain an insight into the diversification and toxin profile associated with *BEAS* genes in *Trichoderma* and *Fusarium* genera.

## 2. Materials and Methods

### 2.1. Fungal Strains, Media and Growth Conditions

All seventeen *Fusarium* and *Trichoderma* strains (Table 1) investigated in this study were characterized earlier [28,29,30,31,32] and deposited in the fungal strain collection of the Institute of Plant Genetics, Polish Academy of Sciences, Poznań, Poland. Purified mycelia of individual fungi were cultivated on plates with potato dextrose agar medium (PDA, Oxoid, Basingstoke, UK) and after seven days collected for genomic DNA extraction. For quantitative beauvericin analysis, fourteen-day-old pure rice cultures of each fungal species were prepared in three replications [22].

### 2.2. DNA Extraction, Molecular Identification, PCR Primers, Cycling Profiles and DNA Sequencing

The genomic DNA extraction was carried out using a modified method with hexadecyltrimethylammonium bromide (CTAB), according to Gorczyca et al. [33]. The fungal identification was performed on the basis of the sequence analysis of a variable fragment of the translation elongation factor 1α gene (*tef*-1α). The beauvericin synthase gene (*BEAS*) was partially amplified using a BEA_F2/BEA_R2 degenerate primer pair designed on basis of a *Fusarium* enniatin/beauvericin synthases sequence, as well as the more distant homologs available in the annotated genomes of *Trichoderma atroviride* and *T. virens*. The resulting marker targeted the conserved methyltransferase domain nested between the 8th and 9th core motifs of the adenylation domain (second functional module). All primers are described in Table 2.

Polymerase chain reactions (PCRs) were carried out using Phire II HotStart Taq DNA polymerase (Thermo Scientific, Espoo, Finland). The conditions for PCR amplification were described earlier by Tomczyk et al. [34].

For sequence analysis, PCR-amplified DNA fragments were purified with exonuclease I (Thermo Scientific, Espoo, Finland) and FastAP shrimp alkaline phosphatase (Thermo Scientific, Espoo, Finland); afterwards they were labeled using forward primer and the BigDyeTerminator 3.1 kit (Applied Biosystems, Foster City, CA, USA) and subsequently precipitated with 96% ethanol. according to Kozłowska et al. [35].

### 2.3. Sequence Analyses and Phylogeny Reconstruction

PCR amplicons were sequenced on Applied Biosystems 3130 apparatus. In order to validate amplicon correctness, the sequences were checked against the reference GenBank sequences of *Hypocreales* fungi (BLASTN with default settings).

CLUSTALW was used to align the sequences [38], and subsequently all gap-containing positions were curated and phylogeny reconstructed using MEGA7 software [39] (maximum parsimony approach, enabled closest neighbor interchange heuristics with default settings, 1000 bootstrap iterations).

### 2.4. Mycotoxin Analyses

#### 2.4.1. Chemicals

A Mili-Q system (Milipore, Bedford, MA, USA) was used to supply water for experiments; all required chemicals for LC-MS analysis were obtained from Sigma-Aldrich (St. Louis, MO, USA) including the beauvericin mass standard (>99%).

#### 2.4.2. Extraction, Purification and Liquid Chromatography Mass Spectrometry Analyses

Beauvericin from pure rice cultures (15 g) of each fungal species was extracted and purified according to previous research conducted by Stępień and Waśkiewicz [31]. The final methanolic solution (2 mL) was filtered using a 0.20 µm Waters HV membrane filter before injection into the UPLC-triple quadrupole mass spectrometer (TQD) system for quantitative analysis. An LC–HRMS/MS spectrum from higher collision dissociation of the [M + Na]^+^ ion and LC-HRMS chromatogram (±5 ppm) of the [M + NH_4_]^+^ ion of beauvericin were presented in previous work [4,40]. Moreover, the fragmentation of the sodiated molecular ion (MS/MS) from beauvericin was performed in full-scan mode (m/z 150–1200), according to Urbaniak et al. [4,40].

The analytical system consisted of the Aquity UPLC chromatograph (Waters, Manchester, MA, USA), electrospray ionization triple quadrupole mass spectrometer (TQD) (Waters, Manchester, MA, USA) in positive mode and chromatographic column Waters Aquity UPLC HSS T3—1.8 µm, 100 × 2.1 mm/ID (Waters, Manchester, MA, USA). Mobile phase compositions were methanol with 0.1% formic acid (line A) and water contained 0.1% formic acid and 2 mM ammonium formate (line B), with the following gradient: from 10 to 90% A in 8 min, then 90% A for 2 min, and return to initial conditions in 2 min. The flow rate was 0.4 mL/min at room temperature, with an injection volume of 3 µL. BEA was identified by comparing the retention time and m/z value obtained by MS and MS^2^ with the mass spectra of the corresponding standard tested under the same conditions (Table 3). For data processing Empower^TM^ 2 software was used (Waters, Manchester, UK).

## 3. Results and Discussion

### 3.1. Fungal Species Identification

Strains representing six different fusarial species, as well as six *Trichoderma* genus members (17 isolates in total) were identified and subject to further analysis. *Fusarium* strains were isolated as plant pathogens from three different host species, and *Trichoderma* strains as saprotrophs from decaying wood (the entire set is summarized in Table 1). Fungi from *Fusarium* genus are cosmopolitan pathogens and possess the ability to colonize a wide range of hosts (e.g., wheat, maize, garlic, asparagus, pineapple) and cause devastating diseases among the plant kingdom [17,22,32,33,41,42,43,44]. In the agricultural context, the most known *Fusarium* diseases are *Fusarium* head blight (FHB), *Fusarium* ear rot, and *Fusarium* wilt, which are difficult to control and generate significant losses in plant production [21,45,46,47,48]. Moreover, fusaria are known to produce numerous, different secondary metabolites, such as the mycotoxins: deoxynivalenol (DON), nivalenol (NIV), zearalenone (ZEN), beauvericin (BEA), enniatins (ENNs), fumonisins B (FBs) and moniliformin (MON). Those toxic compounds are harmful to animals and humans even in low concentrations, may accumulate in plant crops and, thus, are introduced into the food chain in a cascading way [21,49,50,51,52].

In our study, all investigated *Trichoderma* strains were isolated from decaying wood. Most *Trichoderma* spp. are saprotrophic and can colonize a variety of niches, which is greatly facilitated by the synthesis of various lytic enzymes, like cellulases and xylanases [28,53,54,55]. Because of their ability to colonize the rhizosphere and to penetrate the roots, fungi from *Trichoderma* genus as opportunistic symbionts may exert positive effects on plant growth, nutrient assimilation, and systemic resistance through the control of numerous plant-pathogenic fungi, (including *Fusaria*) [56,57,58,59]. They also show antimicrobial or mycoparasitic activities and, to facilitate these activities, produce various active secondary metabolites of potential use as antibiotics or anti-cancer drugs [60,61].

**Figure 2:**
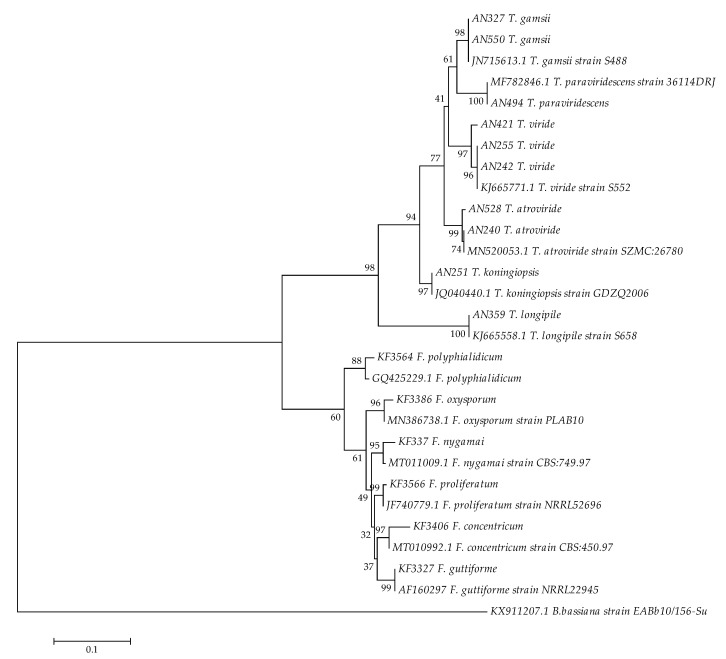
The most parsimonious tree for 6 *Fusarium* and 11 *Trichoderma* strains used in the study, based on the translation elongation factor 1α (*tef*-1α) sequences. *F. concentricum* (GenBank Acc. MT010992.1), *F. nygamai* (GenBank Acc. MT011009.1), *F. oxysporum* (GenBank Acc. MN386738.1), *F. guttiforme* (GenBank Acc. AF160297), *F. polyphialidicum* (GenBank Acc. GQ425229.1), *F. proliferatum* (GenBank Acc. JF740779.1), *T. atroviride* (GenBank Acc. MN520053.1), *T. viride* (GenBank Acc. KJ665771.1), *T. gamsii* (GenBank Acc. JN715613.1), *T. longipile* (GenBank Acc. KJ665558.1), *T. koningiopsis* (GenBank Acc. JQ040440.1), *T. paraviridescens* (GenBank Acc. MF782846.1) and *B. bassiana* (GenBank Acc. KX911207.1) sequences were included as the reference, as well as for outgrouping. The maximum parsimony approach and bootstrap test (1000 replicates) were applied.

Species identification was based on the partial *tef*-1α gene sequences, positioned against the reference sequences deposited in the NCBI GenBank database. All species were identified in agreement with the initial morphological assessment. A sequence from *Beauveria bassiana* (GenBank Acc. KX911207.1) was added to the subsequent phylogenetic analysis as an outgroup (Figure 2). The molecular identification based on the partial *tef*-1α gene has been extensively used in past phylogenetic studies of *Fusarium* and *Trichoderma* species [30,57,62,63,64,65]. Nevertheless, the biosynthetic genes from secondary metabolite gene clusters are receiving attention as reliable, contextual phylogenetic markers e.g., within the producing species complexes [66]. The maximum parsimony reconstruction allowed for the discrimination of the fungal species boundaries and has shown two divided groups of fungi on the dendrogram. In this reconstruction, representative isolates from *Fusarium* genus appear more closely related than strains from *Trichoderma* genus, possible due to different ecological niches/lifestyle preferences covered by the analyzed part of the IPG PAS collection (Figure 2). While the first group is represented by phytopathogenic *Fusarium* strains, the saprotrophic *Trichoderma* strains represented the second group.

### 3.2. Non-Ribosomal Peptide Synthase Genes Divergence

**Figure 3:**
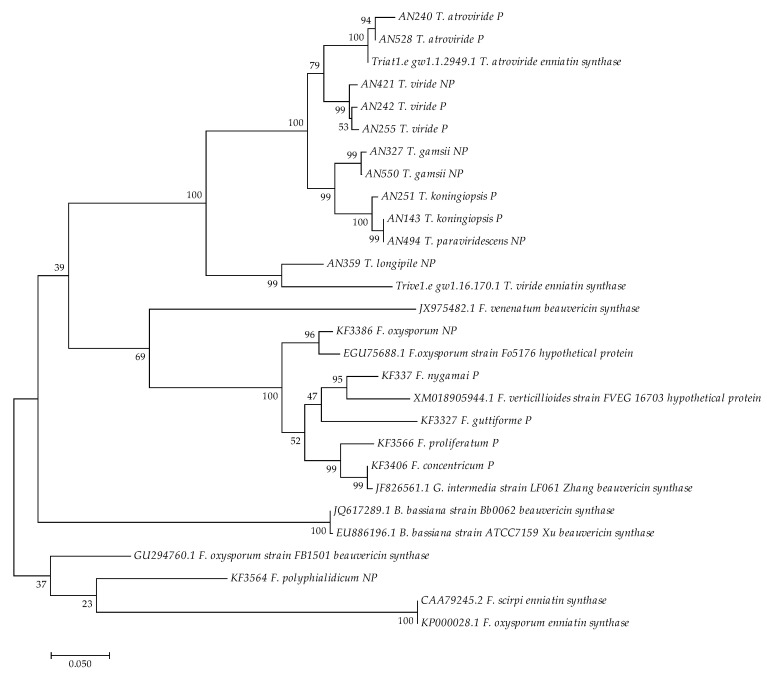
The most parsimonious tree created for a partial beauvericin synthase (*BEAS*) gene sequence obtained with BEA_F2 andBEA_R2 primers from 17 strains of *Fusarium* and *Trichoderma* species. *B. bassiana* (GenBank Acc. EU886196.1; JQ617289.1), *F. proliferatum* (GenBank Acc. JF826561.1 - *G. intermedia*), *F. scirpi* (GenBank Acc. CAA79245.2), *F. venenatum* (GenBank Acc. JX975482.1), *F. verticillioides* (GenBank Acc. XM018905944.1), *F. oxysporum* (GenBank Acc. KP000028.1; GU294760.1; EGU75688.1), *T. atroviride* (JGI ID: Triat1.e_gw1.1.2949.1) and *T. viride* (JGI ID: Trive1.e_gw1.16.170.1) sequences were included as the reference, as well as for outgrouping. The maximum parsimony approach and bootstrap test were applied (1000 replicates). “P” producer or “NP” non-producer of beauvericin.

The sequenced PCR-amplified fragments represented one region of the beauvericin synthase gene and were supplemented by a number of reference cyclic peptide synthase. The entire set was then aligned using the ClustalW algorithm (within MEGA 7 software). The aligned sequences, including partial reference sequences (11 representing *Beauveria*, *Fusarium* and *Trichoderma* genera), trimmed to the amplified region are attached as supplementary material to this work (Figure S1). A maximum parsimony dendrogram was then calculated for the beauvericin synthase (*BEAS*) gene fragments obtained with the BEA_F2/BEA_R2 primers in the different isolates representing both beauvericin-producing and non-producer groups amongst analyzed isolates (Figure 3). While the analysis based on a partial sequence, is by necessity limited, the context of available full length reference sequences (in particular *Trichoderma* sp. exemplars) as well as positioning in the functionally crucial inner fragment of the sequence and the degree of support for the resulting topology is sufficient to support several conclusions.

Three divided groups can be observed, the first one consisting of *Trichoderma* isolates, the second encompassing most fusarial strains and the third comprised of *B. bassiana* and *F. polyphialidicum* (KF3564) strains. The sequences of *Trichoderma* spp. show high nucleotide similarities, between 91% and 100%. In two cases, the obtained sequence fragments suggest that pseudogenisation of synthase has already occurred (frameshift mutations in the amplicons from non-producing isolates—AN 421 and AN 359) The sequences of *Fusarium* spp. show some variance, however the amplicons obtained from *F. proliferatum* (KF3566) and *F. concentricum* (KF3406) strains showed high similarity at about 99% of identical bases. The Genbank-derived sequence of *BEAS* from *F. oxysporum* (GU294760.1) showed considerable divergence, grouping with the reference enniatin synthetases from *Fusarium equiseti* and *F. oxysporum f. cucumerinum* (Figure 3). This would suggest that not only are there distinguishable alleles of *BEAS* and *ESYN* but also that both variants can be found in the strains of the same species. Additionally, based on the phylogeny, in some cases, the earlier similarity-based annotations of synthase products referenced in sequence databases, might need a revision.

The hypothesis of coexisting ENNS/BEAS presence is in line with the findings described in the previous work of Stępien and Waśkiewicz [31]. In that study, Esyn1/Esyn2 and beas_1/beas_2 primers were used to obtain sequences of two different regions of the enniatin synthase gene (*Esyn1*) in various genotypes of *Fusarium* fungi. The phylogenetic analysis clearly showed the divided groups on enniatin and beauvericin producers and revealed that the majority of the strains produced a mixture of BEA and ENNs. In this study, we designed a novel marker based on the availability of *Trichoderma* genome sequences, including beauvericin synthase homologs. We selected the nested methyltransferase domain present in the adenylation domain, in view of its position in the conserved core of the coding sequence as well as mechanical differences between enniatin and beauvericin synthases. In enniatin synthase the A_2_ domains activate and load branched-chain amino acids onto the twin T_2_ domains within module 2, while *BEAS* is specific for phenylalanine and closely integrates with the nested *N*-methyltransferase domain in question [11]. Notably, similar studies were previously performed by Liuzzi et al. [67], where structural determinants in two segments of A_1_ and A_2_ domains were investigated to discriminate *ESYN1* homologs related to the production of enniatins and beauvericin.

Nowadays, multiple partial sequences of the enniatin synthase gene from different fungal species have been published, however, only a few reports are available on the structure of the divergent beauvericin synthase genes [1,31,40,67,68,69,70]. Beauvericin-producing species have been identified by cloning and characterization of the respective biosynthetic genes in *B. bassiana* [11,24], *F. venenatum* [71] and *F. proliferatum* [25]. For *Trichoderma*, an earlier research paper has been published, where authors described the reference sequence as “similar to the *BEAS* gene” based on the pan-genomic analysis (Triat1.e_gw1.1.2949.1, Trive1.e_gw1.16.170.1, utilized in our analyses) [23]. Nevertheless, there is a constant lack of studies involving multiple *Trichoderma* species with respect to this biosynthetic cluster, and there are no reports on beauvericin synthesis by *Trichoderma* sp. available, in conjunction with analysis of the presence of putative synthase homologs. Therefore, the studies on the beauvericin synthase gene cluster are, in our opinion, still informative and worth consideration even in the post-genomic era.

### 3.3. In Vitro BEA Biosynthesis

*Fusarium* fungi are cosmopolitan pathogens and possess the ability to colonize a wide range of crop plants. Moreover, they produce a large number of mycotoxins, including beauvericin, which can contaminate cereal grains, as well as whole plants [21,44,72,73,74]. Therefore, it is essential to study the abilities of these phytopathogens for secondary metabolites production. On the other hand, fungi from the *Trichoderma* genus also appear to produce BEA, despite the fact that they are not phytopathogenic, displaying saprotrophic or endophytic types of growth [75,76]. Amounts of beauvericin produced by investigated fungal strains were measured using the UPLC method, and the results were summarized in Table 4, along with standard deviations calculated for the results obtained for three replicates of each fungal culture. Moreover, the LC–HRMS/MS spectrum from higher collision dissociation of the [M + Na]^+^ ion of beauvericin was added to the supplementary data (Figure S2).

The most efficient producers of beauvericin were found among the *Fusarium* species, which was not surprising because of their pathogenic abilities. Two *Fusarium* strains synthesized beauvericin in the highest amounts—*F. proliferatum*/KF3566 (90 μg/g) and *F. nygamai*/KF337 (22.86 μg/g). In rice culture samples of *F. oxysporum* and *F. polyphialidicum* BEA was not detected. This result can be explained by the fact that both *F. oxysporum and F. polyphialidicum* can change the niches between plant and soil to become non-pathogenic fungi [77,78,79,80]. Only six out of eleven investigated *Trichoderma* strains produced beauvericin in minor amounts on this particular substrate: *T. atroviride*/AN240 (8.78 µg/g), *T. viride*/AN255 (3.02 µg/g), *T. koningiopsis*/AN251 (3.85 µg/g), *T. koningiopsis*/AN143 (4.22 µg/g), *T. viride*/AN242 (2.74 µg/g) and *T. atroviride*/AN528 (5.54 µg/g). The results of low BEA production by *Trichoderma* spp. can suggest that these fungi are characterized by the opportunistic and non-pathogenic style of life [81,82]. It has been shown that *Trichoderma* fungi possess the ability to form mutualistic endophytic relationships with plants, and in this case the production of threatening mycotoxin is not needed and may even be suppressed.

Fungi from the *Trichoderma* genus have been investigated as promising biocontrol agents against *Fusarium* species. *Trichoderma* may suppress *Fusarium* growth in the plant and affect mycotoxin production by these phytopathogenic fungi [57,83,84]. Błaszczyk et al. [57] noticed that three different *Trichoderma* strains (*T. atroviride*, *T. koningiopsis* and *T. viride*) decreased beauvericin production by *F. temperatum*. On the other hand, Rojo et al. [84] did not detect significant differences in BEA production between the control culture—the *F. proliferatum* strain alone and when *T. harzianum* or *T. longibrachiatum* strains were added as antagonists. This contrast in observations can be explained by the complexity of factors that may have an influence on beauvericin biosynthesis. Fungicides, weather, and the type of substrate or growing conditions used can have an impact on BEA production [20,72]. Epigenetic or genetic changes such as pseudogenization of a silent cluster can occur gradually or rapidly (e.g., via the putative frameshift mutations observed during our analysis), which in future work can be investigated via profiling of transcript and protein product expression. In the context of plant disease it is worth noting that while beauvericin is known to be a virulence factor in entomopathogenicity and not phytopathogenicity, the ubiquity of certain fusaria as endophytes (e.g., [85]) and possibility of opportunistic entomopathogenicity ([86]) in the genus suggests that the production of beauvericin could allow for successful competition with other organisms (insects, fungi and bacteria) in a plant-associated context in the absence of toxicity to host plants.

Additionally, it is important to note that the amount of beauvericin in the substrate may depend on its composition. BEA is easily dissolved in water and degraded by fungal enzymes, with likely resistance in producers/former producers. Moreover, it can be modified towards various derivative/similar compounds due to the enzyme’s low substrate specificity towards branched-chain amino acids and the relative abundance of the enzyme’s substrates in the cellular pool [87]. Nowadays, over 17 various beauvericin analogs have been described differing in chain composition [4,40]. Beauvericin analogs may form naturally or by adding the amino acid precursor to the medium. The possibility of biotransformation by an unrelated pathway is likewise to be considered [4,5,6,7,8,40]. Although environmental, biological, and chemical factors are important in the regulation of beauvericin synthesis, genetic determinants play a critical role. Not only are primary genes encoding the enzymes participating in beauvericin formation necessary, but accessory genes are also essential to deliver the precursors to the main process, thus, affecting the final composition of the product [67].

## 4. Conclusions

This study showed the differences in beauvericin production by a number of distant relatives—fungal species belonging to *Fusarium* and *Trichoderma* genera. We also highlighted the high variance of the non-ribosomal peptide synthase gene sequence among individual fungal species from the *Hypocreales* order and possible pseudogenization of core synthase gene in some non-producers. Further studies are required to explore the basis of the differences in BEA synthesis inside the *Trichoderma* genus in order to explain if the ability to synthesize beauvericin is essential for these saprotrophic fungi.

## Figures and Tables

**Table 1:** Characterization of studied *Fusarium* and *Trichoderma* strains.

Species	Strain	Source/Host	References
*T. atroviride*	AN240	decaying wood	[28]
*T. viride*	AN255	decaying wood	[29]
*T. koningiopsis*	AN251	decaying wood	[28]
*T. koningiopsis*	AN143	decaying wood	[30]
*T. viride*	AN242	decaying wood	[28]
*T. gamsii*	AN327	decaying wood	[28]
*T. longipile*	AN359	decaying wood	[28]
*T. viride*	AN421	decaying wood	[28]
*T. atroviride*	AN528	decaying wood	Present study
*T. paraviridescens*	AN494	decaying wood	[28]
*T. gamsii*	AN550	decaying wood	[30]
*F. proliferatum*	KF3566	*Oryza sativa*	[31]
*F. oxysporum*	KF3386	*Ananas comosus*	[32]
*F. concentricum*	KF3406	*Ananas comosus*	[32]
*F. polyphialidicum*	KF3564	*Ananas comosus*	[32]
*F. nygamai*	KF337	*Cajanus indicus*	[31]
*F. guttiforme*	KF3327	*Ananas comosus*	[32]

**Table 2:** PCR primers used in this study.

Marker	5′ > 3′ Sequence	Temperature of Annealing (°C)	Amplicon Size (bp)	Reference
Ef728M TefR1	CATCGAGAAGTTCGAGAAGG GCCATCCTTGGAGATACCAGC	63	600	[36,37]
BEA_F2 BEA_R2	TGGACDTCHATGTAYGAYGG GGCTCRACRAGMARYTCYTC	61	570	Present study

**Table 3:** Parent and daughter ions, collision energy and limit of detection (LOD) and quantification (LOQ) (ng/g) for beauvericin.

Compound	Parent Ion (m/z) [M+NH_4_]^+^	Primary Daughter Ion (m/z)	Secondary Daughter Ion (m/z)	Collision Energy (eV)	LOD ^a^ (ng/g)	LOQ ^b^ (ng/g)
BEA	801.2	784.0	244.1 *	28	1	3

* Transitions used for quantification. ^a^ Limit of detection (LOD). ^b^ Limit of quantification (LOQ).

**Table 4:** Mean concentrations with standard deviations of beauvericin (μg/g) produced in vitro by studied *Fusarium* and *Trichoderma* strains.

Species	Strain	Concentration of Beauvericin [µg/g]	References
*T. atroviride*	AN240	8.78 ± 0.92	Present study
*T. viride*	AN255	3.02 ± 0.41	Present study
*T. koningiopsis*	AN251	3.85 ± 2.77	Present study
*T. koningiopsis*	AN143	4.22 ± 0.39	Present study
*T. viride*	AN242	2.74 ± 0.35	Present study
*T. gamsii*	AN327	ND	Present study
*T. longipile*	AN359	ND	Present study
*T. viride*	AN421	ND	Present study
*T. atroviride*	AN528	5.54 ± 0.46	Present study
*T. paraviridescens*	AN494	ND	Present study
*T. gamsii*	AN550	ND	Present study
*F. proliferatum*	KF 3566	90.85 ± 10.21	[31]
*F. oxysporum*	KF 3386	ND	[32]
*F. concentricum*	KF 3406	0.51 ± 0.06	[32]
*F. polyphialidicum*	KF 3564	ND	[32]
*F. nygamai*	KF 337	22.86 ± 2.66	[31]
*F. guttiforme*	KF 3327	7.70 ± 1.15	[32]

## Supplementary Materials

The following are available online at https://www.mdpi.com/2309-608X/6/4/288/s1, Figure S1: The 17 aligned sequences of investigated Fusarium and Trichoderma strains with partial reference sequences, trimmed to the amplified region. Figure S2: LC–HRMS/MS spectrum from higher collision dissociation of the [M + Na]^+^ ion of beauvericin.
